# Spontaneous and tetracosactide‐induced anti‐ACTH antibodies in man

**DOI:** 10.1111/cen.12795

**Published:** 2015-06-25

**Authors:** Earn H. Gan, Katie MacArthur, Anna L. Mitchell, Abhijit Joshi, Patricia Crock, Simon H. S. Pearce

**Affiliations:** ^1^ Institute of Genetic MedicineNewcastle UniversityNewcastle upon TyneUK; ^2^ Endocrine UnitRoyal Victoria InfirmaryNewcastle upon TyneUK; ^3^ Cellular Pathology DepartmentRoyal Victoria InfirmaryNewcastle upon TyneUK; ^4^ John Hunter Children's HospitalUniversity of NewcastleNewcastleNSWAustralia

## Abstract

**Context:**

During a clinical trial of regular tetracosactide depot injections, four of 13 patients with autoimmune Addison's disease (AAD) developed adverse reactions immediately following tetracosactide injections. We wished to investigate whether these adverse effects could be due to the production of circulating antitetracosactide (ACTH_1–24_) antibodies.

**Design:**

Anti‐ACTH binding activity was investigated using immunoblotting and ELISA on sera from participants in the trial (*n* = 13; baseline and after tetracosactide exposure), 131 unrelated patients with AAD, 92 patients with Graves’ disease (GD), 15 patients with isolated ACTH deficiency and 102 controls. Immunohistochemistry of human pituitary tissue sections was also performed using pooled sera.

**Results:**

Bands at approximately 4 and 6 kDa, corresponding to ACTH_1–24_ and full‐length ACTH_1–39,_ respectively, were found in 10 of 13 (77%) of sera from trial patients exposed to tetracosactide, including all those who had an adverse reaction. This is in contrast with healthy control sera, which showed no binding. The same 10 subjects also showed high levels of binding to tetracosactide by ELISA, along with 21% of patients with AAD, 14% of patients with GD (both *P* < 0·001 compared to controls) and 1 isolated ACTH deficiency patient (7% of 15). These sera also recognized native ACTH in human pituitary sections.

**Conclusion:**

Our study demonstrates that repeated administration of depot tetracosactide can lead to anti‐ACTH_1–24_ autoreactivity. In addition, a significant number of patients with AAD and GD also had similar, spontaneous, anti‐ACTH reactivity. The presence of these antibodies could mediate some of the adverse effects or explain the well‐described phenomenon of resistance to chronic ACTH therapy.

## Introduction

The immune system is programmed to discriminate between self‐ and non‐self‐peptides, with the aim of eliminating foreign organisms and proteins that are harmful, but without generating an immune response to endogenous self‐peptides. Nevertheless, tolerance to endogenous self‐proteins can break down to produce autoimmune disorders, in which immune responses may be directed against many different proteins, most typically enzymes (e.g. thyroid peroxidase, steroid 21‐hydroxylase, glutamic acid decarboxylase) or cell‐surface receptors (e.g. TSH receptor, acetylcholine receptor and calcium‐sensing receptor).^1^, ^2^, ^3^, ^4^, ^5^ In addition, a lesser number of conditions are characterized by antibodies against secreted peptide hormones, the most common of which is type 1 diabetes in which anti‐insulin antibodies are highly prevalent.^6^, ^7^

In a recent clinical trial, we administered high‐dose synthetic adrenocorticotrophic hormone (ACTH_1–24_ as zinc tetracosactide; depot synacthen) to patients with autoimmune Addison's disease (AAD) in an attempt to stimulate adrenal regeneration (RoSA study).^8^ However, towards the end of the study, four of 13 patients with AAD (all females) developed adverse reactions immediately after tetracosactide injections during an ACTH_1–24_ stimulation test, despite having tolerated multiple depot tetracosactide injections. One patient experienced generalized weakness, heavy legs, nausea and abdominopelvic pain 5 min after tetracosactide injection, which resolved after 70 min. Another patient felt nauseous and lost consciousness for a few seconds, with transient hypotension and abdominal cramps. Two other female participants developed wheals at previous tetracosactide injection sites, with itchiness and redness over their palms and soles. One of the latter also experienced lower abdominal cramps and vomiting, followed by intermenstrual spot bleeding for 24 h. Furthermore, one participant whose adrenal function initially improved during ACTH_1–24_ therapy failed to sustain the clinical improvement. Despite the fact that ACTH is an endogenous peptide to which immune tolerance should be established, these phenomena made us wonder whether there were antibodies generated against the depot synacthen therapy.

ACTH is a polypeptide composed of 39 amino acids.^9^ The amino N‐terminal segment of the peptide (residues 1–24) is the biologically active region of ACTH and is highly conserved across vertebrate species, whereas the carboxyl terminal is considered to be more antigenic.^10^ Tetracosactide, also known as cosyntropin or synacthen, is a synthetic polypeptide comprising the amino‐terminal 24 amino acids of ACTH. Depot tetracosactide used in our clinical study has a prolonged duration of action compared to soluble, unconjugated tetracosactide, as the peptides are absorbed onto a zinc phosphate substrate, delaying its absorption from the site of injection. In this study, we investigated the possibility that immunoreactivity towards tetracosactide therapy developed in our trial patients, and we studied this in additional patient cohorts.

## Methods

### Patients

Thirteen Caucasian patients with established AAD were recruited for a clinical study entitled the revival of steroidogenic function in AAD (RoSA; NCT01371526), either from the endocrine clinics of the Newcastle upon Tyne NHS hospitals (*n* = 12), or from self‐referred following an ethics committee agreed advertisement in the national Addison's disease self‐help group (ADSHG) quarterly newsletter (*n* = 1). Clinical details of these patients and the trial have been provided elsewhere.^8^

The sera from 131 patients with AAD and 92 patients with Graves’ disease were obtained via recruitment since 1996 through outpatient endocrinology services in the North East of England and the UK Addison's disease self‐help group. Sera from 102 patients with negative autoimmune screening (rheumatoid factor, antinuclear antibody, antimitochondrial antibody, and anti‐gastric cell antibody) were obtained from the Clinical Pathology department at Newcastle upon Tyne NHS hospitals trust. These sera were used as ‘nonautoimmune’ controls. Fifteen Caucasian patients with isolated ACTH deficiency were recruited either from the John Hunter Children's Hospital, Newcastle Australia (*n* = 11) or from Newcastle upon Tyne NHS hospitals (*n* = 4). This study was carried out with approval of the North East – Sunderland Research Ethics Committee (ref. 12/NE/0101) and the Hunter New England Human Research Ethics Committee.

### Detection of antisynacthen antibodies by immunoblotting

A 16% polyacrylamide tricine gel was run (90V, 1 h) with 2·5 μg of tetracosactide per well and was electroblotted (350 mA, 45 min) onto Hybond C membrane (Amersham). Patient and control sera were diluted 1:250 and incubated for 75 min at room temperature, following blocking with 5% nonfat milk powder. Following washing, goat anti‐human IgGγ‐chain‐horseradish peroxidase (HRP) (A6029; Sigma‐Aldrich, Poole, UK) was used to oxidize diaminobenzidine (DAB) substrate (Sigma‐Aldrich). Additional blots made using full‐length human ACTH_1–39_ (Sigma‐Aldrich) were probed, and a monoclonal anti‐ACTH_1–24_ antibody (1B55; Santa Cruz, Dallas, CA, USA) was used as a positive control (detected with goat antimouse IgG (A4416, Sigma‐Aldrich).

### Detection of antitetracosactide antibodies by enzyme‐linked immunosorbent assay (ELISA)

Tetracosactide (ACTH_1–24_) was bound to the solid phase of ELISA plates (NUNC maxisorp; Thermo Fisher Scientific, Waltham, MA, USA) in a concentration of 1 μg/ml overnight at 4 °C in 1× carbonate coating buffer. Plates were washed three times in phosphate‐buffered saline Tween 20 (PBST; PBS, 1% Tween 20) and blocked with 2% bovine serum albumin (BSA) for 1 h at room temperature. Sera diluted to 1:250 in 2 mg/ml BSA were incubated in plates at room temp for 75 min, followed by washing and secondary antibody (goat anti‐human IgG γ‐chain‐HRP) diluted 1:500 in 2 mg/ml BSA for 1 h at room temperature. Following washing and detection with 3,3′,5,5′‐tetramethylbenzidine (TMB) substrate (T0440; Sigma‐Aldrich) for 3 min, reactions were stopped by 1M hydrochloric acid, and absorbance measured at A_450_ on an Ascent Multiskan (Thermo Fisher Scientific). Antitetracosactide binding activity was measured in triplicate for each participant sera, and each result reproduced on at least two different days. Results are expressed as arbitrary units of absorbance, having subtracted the background absorbance (mean of three wells without serum). The patient's serum that showed the strongest antisynacthen activity was selected as the positive control (serum of participant 6 at week 20, dilution at 1:250 in 2 mg/ml BSA). The positive control was run on each occasion to check for interassay variation, which was within 10% on successive days. To check for nonspecific serum binding to peptides, all positive samples were run with an identical ELISA protocol, with plates coated with octreotide (somatostatin 1–8) 1 μg/ml instead of tetracosactide.

### Immunohistochemistry study of pituitary tissues

Human pituitary tissues fixed with 4% paraformaldehyde and embedded in paraffin were sectioned in 4 μm sections. They were immunostained using the indirect HRP‐labelled antibody method. Sections underwent deparaffinization and rehydration. Antigens were retrieved by boiling the sections in Tris–EDTA buffer (10 mm Tris Base, 1 mm EDTA solution, 0·05% Tween 20, pH 9·0) for 10 min in a microwave (850W). The sections were allowed to cool for 20 min followed by two washes with PBS plus 0·025% Triton X‐100. The sections were then blocked with 10% goat serum in 1% BSA for an hour. This was followed by 4 °C overnight incubation with pooled patient sera (from three positive subjects) diluted 1:100. Following two washes with PBS plus 0·025% Triton X‐100, sections were incubated in 1% hydrogen peroxide for 15 min. The slides were then washed twice prior to incubation with HRP conjugated goat anti‐human IgGγ‐chain antibodies (A6029; Sigma‐Aldrich) for 1 h. Antibody binding was visualized with the chromogen diaminobenzidine (DAB) (Sigma‐Aldrich) and counterstained with haematoxylin. Monoclonal anti‐ACTH_1–24_ antibody (1B55; Santa Cruz) was used as a positive control (detected with goat antimouse IgG (A4416; Sigma‐Aldrich).

## Results

### Antitetracosactide immunoreactivity among patients in the RoSA study

**Figure 1:**
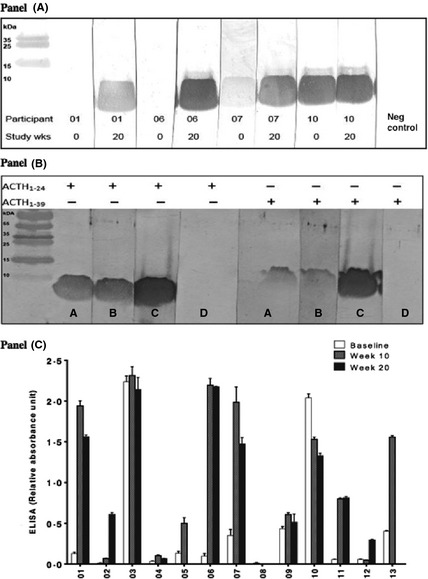
Antitetracosactide immunoreactivity detected with immunoblotting and ELISA. Panel A: Detection of serum binding activity to immunoblotted ACTH1–24, at baseline and week 20 for participants 1, 6, 7 and 10, as assessed by the band of approximately 4 kDa, the expected molecular weight of ACTH1–24. Sera from healthy subjects were used as a negative control. Panel B: Immunoblotting was performed with blots made using both tetracosactide (ACTH1–24) peptide and full‐length human ACTH1–39, both 2·5 μg per well. Representative positive sera from two participants (sera A and B), a monoclonal anti‐ACTH1–24 antibody (C) and serum from a healthy control (serum D) are shown. The monoclonal anti‐ACTH antibody recognizes bands of the same molecular mass as the positive participant sera for both ACTH1–24 and ACTH1–39 peptides, which was not seen with control sera. Panel C. Anti‐tetracosactide binding activity on immunoblotting from all the patients of RoSA study was quantified using ELISA. Results (±SEM) are expressed as arbitrary units of absorbance at 450 nm, having subtracted the background absorbance (mean of three wells without serum). A total of 11 females and two males (04 and 08) participated in this study. All except five patients had relative absorbance units of less than 0·21 at baseline. A positive control was run on each occasion to check for interassay variation, which was within 10% on successive plates.

Sera from all of the patients who had an adverse reaction to tetracosactide identified a approximately 4 kDa band on immunoblotting against the tetracosactide (ACTH_1–24_) peptide (Fig. 1 panel A). This band was found in 10 of 13 (77%) patients who had received multiple doses of tetracosactide depot as part of the clinical study. In most patients, the immunoreactivity developed over time during exposure to ACTH_1–24_, but in two of the participants, there was reactivity at baseline before sustained peptide exposure.

Immunoblotting against full‐length ACTH_1–39_ peptide showed that the patient sera which reacted with the tetracosactide peptide also showed specific reactivity at approximately 6 kDa with full‐length ACTH peptide (Fig. 1 panel B). This is in contrast to results from 3 healthy control sera, which showed no binding to either the ACTH_1–24_ or the ACTH_1–39_ peptides. A commercial anti‐ACTH amino‐terminal (corresponding to the tetracosactide peptide) monoclonal antibody was used to probe blots and showed bands corresponding to the expected size with both the tetracosactide and the full‐length ACTH peptides (Fig. 1 panel B). An ELISA was then designed, using tetracosactide peptide captured onto solid phase, to further quantify the antisynacthen binding activity (see below for ascertainment of threshold of normality). Sera from five of the 13 patients (38%; all female) demonstrated relative absorbance above 0·21 units at baseline (the threshold for positivity, see below) with 2 of them having a relative absorbance of more than 2 units (Fig. 1 panel C). Upon completion of either 10 or 20 weeks of tetracosactide therapy, 11 of 13 (84·6%) patients showed immunoreactivity towards tetracosactide, with relative absorbance scores above 0·21 units. Nine of the 13 participants (69%) demonstrated a 1·5‐ to 12‐fold increase in the ELISA absorbance score following tetracosactide peptide treatment, but sera from both male patients were negative throughout the study.

### Antitetracosactide immunoreactivity among controls *vs* patients with autoimmune diseases (autoimmune Addison's disease, Graves’ disease and isolated ACTH deficiency)

**Figure 2:**
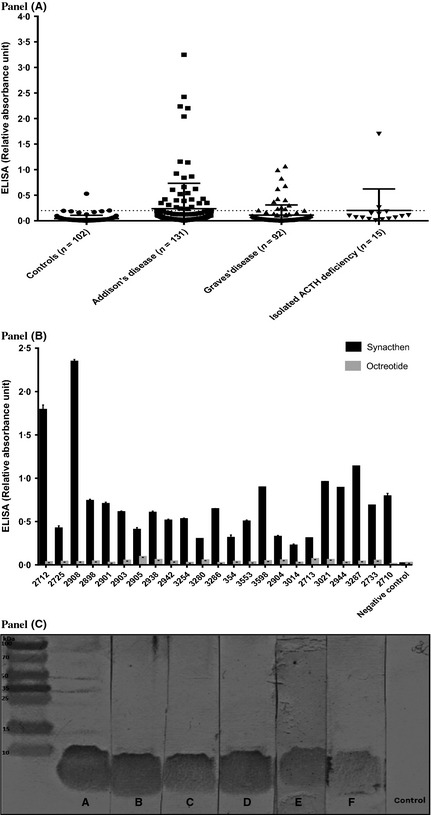
Anti‐tetracosactide binding activity detected by ELISA and immunoblotting. Panel A. Relative quantitation of anti‐tetracosactide binding activity using ELISA with sera from 102 controls, 131 unrelated patients with AAD, 92 patients with Graves’ disease and 15 patients with isolated ACTH deficiency. Tetracosactide (ACTH1–24) 1 μg/ml was bound to solid phase ELISA plates (NUNC maxisorp). Results (±SEM) are expressed as arbitrary units of absorbance at 450 nm, having subtracted the background absorbance (mean of three wells without serum). A positive control was run on each occasion to check for interassay variation, which was within 10% on successive plates. ELISA of the 102 control sera (from anonymous hospital attendees with negative autoantibody status) showed absorbance readings of ≤0·2 units in 101 sera, with 1 serum showing an absorbance of 0·6 units. Panel B. All positive samples were run against an identical ELISA protocol, with plates coated with octreotide (somatostatin 1–8) 1 μg/ml. None of the sera gave a positive signal in the assay with octreotide. Panel C. All positive patient sera in the ELISA were tested against the tetracosactide peptide on immunoblotting, and all 41 showed specific 4 kDa binding. Representative positive sera from six patients (A–F) are shown.

The study of autoimmunity towards ACTH_1–24_ or ACTH_1–39_ peptide was extended to larger groups involving healthy controls, patients with Graves’ disease, autoimmune Addison's disease and isolated ACTH deficiency. Initially, 102 control sera from anonymous hospital attendees with negative autoantibody status were investigated, along with a patient serum that had proven positive on immunoblotting. Only one of the 102 control sera exceeded 0·21 absorbance units in the ELISA (Fig. 2, panel A). This value was then taken as an arbitrary threshold of positivity. Of the sera from 131 patients with autoimmune Addison's disease, 28 (21%) were positive (>0·21 U) for antitetracosactide binding on the ELISA assay. Similarly, sera from 92 patients with Graves’ disease and 15 with isolated ACTH deficiency were run: 13 (14%) and 1 (7%) were positive, respectively.

To investigate whether the antisynacthen peptide binding in the ELISA could be attributable to nonspecific binding, all positive sera were tested in an identical assay, except that the plates were coated with octreotide (somatostatin octapeptide) instead of tetracosactide. None of the sera gave a positive signal in the assay with octreotide (Fig. 2, panel B). Similarly, all positive patient sera in the ELISA were tested against the tetracosactide peptide on immunoblotting, and all 41 showed specific 4 kDa binding (Fig. 2, panel C).

### Immunohistochemistry study

**Figure 3:**
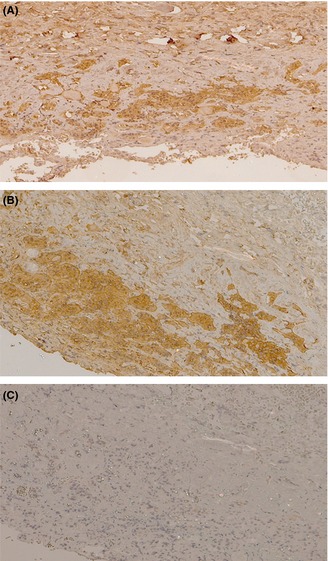
Cytoplasmic anti‐ACTH1–24 staining of human anterior pituitary sections. Immunohistochemistry was performed to identify whether sera with anti‐tetracosactide binding activity would recognize native ACTH in contiguous tissue sections. Representative positive patient sera (panel A) and negative patient sera (panel C) were used. Monoclonal anti‐ACTH1–24 antibody (1B55; Santa Cruz) was used as positive control (panel B). The positive sera produced a cytoplasmic staining in a similar pattern to that of the commercial anti‐ACTH antibody.

Finally, we examined normal human pituitary sections by immunohistochemistry to determine whether the patient sera with antisynacthen binding activity would recognize native ACTH in tissue sections. Figure 3 shows that these positive sera produced cytoplasmic staining in a similar pattern to that of a commercial anti‐ACTH antibody.

## Discussion

The development of serum anti‐ACTH antibodies following long‐term porcine or synthetic full‐length ACTH peptide therapies was reported in the 1960s and 1970s.^11^, ^12^ Glass and colleagues detected anti‐ACTH antibodies using haemagglutination and antigen displacement methods in 32% of 38 rheumatoid arthritis subjects treated with long‐term depot tetracosactide (zinc tetracosactide).^13^ In line with these early observations, we demonstrated the development of anti‐tetracosactide and full‐length ACTH binding activities using immunoblotting among 77% of 13 patients with autoimmune Addison's disease, treated with 20 weeks of depot tetracosactide therapy. The concentration of these anti‐ACTH antibodies also appeared to increase in most, but not all subjects, with increasing time of exposure to depot tetracosactide therapy.

Although antibodies to self‐peptides should in theory not occur, the development of antibodies to various peptide therapies has been extensively reported. Anti‐insulin antibodies were observed in patients with diabetes treated with exogenous insulin, leading to immunological resistance and poor glycaemic control.^14^, ^15^ A similar experience was found in patients treated with growth hormone therapy and with enzyme replacement in Gaucher's disease.^16^, ^17^, ^18^ Hence, our finding is in keeping with this phenomenon and may explain some of the side effects seen in the RoSA study. In addition, modification of circulating tetracosactide concentrations by sequestration or increased clearance mediated by circulating anti‐ACTH antibodies could account for treatment failure in some of the patients and ‘treatment resistance’ observed in one participant, who lacked a sustained improvement during tetracosactide therapy. Indeed, Felber and colleagues described inactivation of corticotropin following antibody formation towards porcine ACTH, using a radioimmunoassay.^11^ It has also been shown that antibodies generated in guinea pigs immunized with synthetic full‐length ACTH were responsible for a loss of ACTH bioactivity *in vitro*.^11^ Furthermore, as antibodies against tetracosactide would be directed against the biologically active amino acid sequence of ACTH, the 1–24 segment, this clearly has the potential to reduce the therapeutic potency and effectiveness of depot tetracosactide therapy.

Intriguingly, the same autoreactivity has not been reported with soluble synthetic tetracosactide. Indeed, animal studies showed that whilst conjugates of the ACTH_1–24_ peptide were antigenic, unconjugated ACTH_1–24_ peptide failed to induce an antibody response in rabbits or guinea pigs.^11^, ^19^ The N‐terminal segment (1–24) of ACTH peptide is highly conserved among all species and is believed to have low antigenicity.^10^, ^12^, ^20^ Hence, it seems likely that the complex of ACTH_1–24_ and zinc in the depot tetracosactide formulation preparation may be important in the generation of the humoral immune response.

Interestingly, five of the 13 participants from the RoSA study, who were never previously exposed to depot tetracosactide, demonstrated positive serum anti‐ACTH binding activity at baseline. Similar findings were noted in the larger cohort of patients with Addison's. These latter patients had all received one or two soluble ACTH_1–24_ peptide injections during diagnostic testing for Addison's disease. Hence, we speculate that the anti‐ACTH antibody response could potentially have been generated from either previous exposure to soluble tetracosactide, or more likely towards native, full‐length endogenous corticotropin. This group of patients may be more susceptible to the breakdown of immune self‐tolerance, by dint of their autoimmune Addison's disease, but may also be susceptible owing to the very high levels of endogenous plasma ACTH circulating in the months before their diagnosis of AAD. Indeed, several previous studies have found an activity in the sera of patients with AAD that appeared to attenuate the *in vitro* steroidogenic response of cultured adrenal tissue to ACTH.^21^, ^22^, ^23^ Although this was believed to be due to patient immunoglobulins that bound to and blocked stimulation of the ACTH receptor, several of the effects described would be consistent with antibodies that neutralized ACTH action *in vitro*. This then begs the question, could these ACTH antibodies that were found in 21% of the unrelated patients with AAD in our cohort, contribute to the pathogenesis of autoimmune Addison's disease? If these antibodies had functional consequences in terms of binding or clearing endogenous ACTH, then a state of relative ‘corticotropin resistance’ could potentially lead to an accelerated decline in adrenal function during the natural history of evolving Addison's disease. Plasma ACTH may be elevated for many years prior to the diagnosis of Addison's disease^24^ and this ACTH drives small islands of adrenal hyperplasia and regeneration,^25^, ^26^ slowing the ultimate onset of adrenal steroidogenic failure. Thus, early adrenal unresponsiveness to ACTH stimulation in evolving Addison's disease may have the potential to tilt the balance from subclinical or partial adrenal insufficiency to clinically apparent adrenal insufficiency.

The significance of anti‐ACTH antibodies in a small number of patients with Graves’ disease is also unknown, especially in the absence of clinically apparent adrenal insufficiency. Nevertheless, several studies have documented a blunted cortisol response to tetracosactide in hyperthyroid Graves’ disease, which would be consistent with the effects of anti‐ACTH antibodies in these patients.^27^, ^28^ It is also well known that patients suffering from one autoimmune disease are susceptible to development of a second autoimmune condition, manifesting either as overt disease following pathological destruction of self‐tissue or as the presence of circulating autoantibodies alone.

Following the demonstration that sera with anti‐ACTH antibodies recognized native ACTH in human pituitary sections (Fig. 3), we also wondered whether anti‐ACTH antibodies could have a role in isolated ACTH deficiency, as previously suggested in a single case report.^29^ On account of the rarity of this condition, we were able to obtain sera from just a small number of individuals, and only one serum sample showed anti‐ACTH binding activity. Whilst isolated ACTH deficiency is a heterogeneous condition, a significant proportion of individuals have coexisting autoimmune thyroid disease or manifest other features of autoimmunity, and the target autoantigen has yet to be defined. Nevertheless, the presence of anti‐ACTH antibodies in just one (7%) of our patient group suggests that although these antibodies would be a logical mediator of isolated ACTH deficiency, they are not a major cause of this condition. Larger cohorts of this rare condition should be examined in the future to confirm our preliminary findings.

In summary, this study demonstrates that repeated administration of depot tetracosactide can lead to anti‐ACTH autoreactivity. In addition, a significant number of untreated patients with AAD and GD also had similar autoreactivity (*P* < 0·001 *vs* controls). It is possible that these anti‐ACTH antibodies mediated some of the adverse effects seen in participants in the RoSA study and that their generation in individuals following multiple ACTH injections could explain the well‐described phenomenon of resistance to chronic ACTH therapy. In the future, further investigation is necessary to more fully determine the role of these anti‐ACTH antibodies in the pathogenesis of autoimmune Addison's disease, isolated ACTH deficiency and other autoimmune conditions.

## Competing interests

SP has received speaker fees from Merck‐Serono and Viropharma. No other author declares any conflict of interest.
